# The National Medical Union

**Published:** 1914-01-10

**Authors:** Robert Carswell, E. Arthur Dorrell

**Affiliations:** Hon. Secretary; Hon. Secretary


### THE PROFESSION OF MEDICINE.

### The National Medical Union.

Meetings of the executive, press, and finance committees will be held in the offices of the Union, 346 Strand, on Saturday, January 17, at 4.30 p.m., to consider the steps to be taken to enrol all non-panel members of the profession who are in sympathy with the objects and policy of the Union, together with other business.

The publication in the press of a preliminary statement of the objects and policy of the Union has attracted considerable attention in London, Edinburgh, Glasgow, Manchester, Liverpool, and other parts of the country. Letters of application for membership and for particulars, which have subsequently been received from medical men in Canterbury, Peterborough, Staffordshire, Dawlish, Ilford, Leyton, Derby, Cambridge, Southport, Cheshire, and elsewhere, show that a lively interest is being taken by the profession generally in the movement, Liverpool being especially active in the cause.

Robert Carswell,
E. Arthur Dorrell,
Hon. Secretaries.

